# Functional Separators for Long-Life and Safe Li Metal Batteries: A Minireview

**DOI:** 10.3390/polym14214546

**Published:** 2022-10-26

**Authors:** Yanyan Li, Yu Zhao, Yong Yang, Zhijie Wang, Qin Yang, Jiaojiao Deng

**Affiliations:** 1 State Key Laboratory of High-Performance Ceramics and Superfine Microstructures, Shanghai Institute of Ceramics, Chinese Academy of Sciences, 1295 Dingxi Road, Shanghai 200050, China; 2 Key Laboratory of Advanced Carbon Materials and Wearable Energy Technologies of Jiangsu Province, Soochow Institute for Energy and Materials Innovations, Light Industry Institute of Electrochemical Power Sources, College of Energy, Soochow University, Suzhou 215006, China; 3 Department of Applied Physics, The Hong Kong Polytechnic University, Hung Hom, Hong Kong, China; 4 Shenzhen Geim Graphene Center, Shenzhen Key Laboratory on Power Battery Safety, Tsinghua Shenzhen International Graduate School (SIGS), Shenzhen 518071, China

**Keywords:** li metal batteries, functional separators, high safety

## Abstract

Lithium (Li) metal batteries (LMBs) have received extensive research attention in recent years because of their high energy density. However, uncontrollable Li dendrite growth deteriorates the battery life and brings about severe safety hazards. The rational design of battery separators is an effective approach to regulate uniform Li metal deposition towards boosted cycle life and safety of LMBs. Herein, we review the recent research progress concerning this issue, including mechanically strengthened separator fabrication, functional separator construction towards regulated Li ion deposition, and flame-retardant separator design. Moreover, the key issues and prospects of optimal design of separators are clarified for future development. This minireview is expected to bring new insight into developing advanced separators for long-life and safe LMBs.

## 1. Introduction

As one of the most mature clean energy storage devices, Li-ion batteries have been widely used in various portable electronic products and electric vehicles [1,2]. Nevertheless, with the urgent need for long cruising range (>300 km) and long service life in electric vehicles, there is an increasing demand for higher energy density in rechargeable batteries [3,4,5]. To achieve this goal, it is of great importance to develop electrode materials with higher capacity. Li metal has an ultrahigh theoretical capacity of 3860 mAh g^−1^, a low mass density of 0.534 g cm^−3^, and an extremely low electrochemical reduction potential (−3.04 V vs. standard hydrogen electrode), and has been regarded as the most promising anode material [6,7]. However, the formation of an unstable solid electrolyte interface (SEI), the large volume change during Li plating/stripping cycles, and the uncontrollable growth of Li dendrites deteriorate the cycle life and safety of Li metal batteries (LMBs), seriously hindering their practical applications [8,9,10].

Various strategies have been proposed to address the above challenges in LMBs, including constructing functional artificial SEI [11,12,13,14], electrolyte engineering [15,16,17], separator modification [18,19,20,21,22,23], solid-state electrolyte design [24,25], and the construction of 3D composite Li metal anodes [26,27,28,29]. Among these strategies, separator modification plays an important role, because it would result in little change in the volume/mass of the battery, and thus would have little effect on the energy density of the Li metal battery. In addition, separators can be prepared at large scale, which helps to reduce the cost. The most widely used separators, such as polyethylene (PE) and polypropylene (PP), have the characteristics of porosity and electrolyte wettability. However, the low melting point of traditional separators leads to weak thermal stability and deterioration of mechanical properties as the temperature exceeds the melting point. Some nanofiber membranes with high porosity and outstanding thermal stability, such as polyacrylonitrile (PAN) and polyvinylidene fluoride (PVDF), can replace the above-mentioned separators, but suffer from poor mechanical strength due to weak physical interaction between the fibers. Therefore, by changing the composition and structure of the separator, the transport path of the Li ion can be adjusted, which would be beneficial in regulating Li nucleation and deposition behaviors. In addition, the functional separators can improve the mechanical strength to suppress Li dendrite growth and prevent piercing caused by Li dendrites.

The designing and optimization of separators, including modifying separators with functional polymers [30,31], carbon materials [32,33], metal particles [34,35], and solid electrolytes [36,37], have been widely studied. Given the increasing research activity on the optimal design of Li battery separators in recent years, a timely and comprehensive review of this interesting and sustainable research area is highly desirable. Here, in this minireview, we discuss the recent research progress of mechanically strengthened separator fabrication, functional separator construction towards regulated Li ion deposition, and flame-retardant separator design. Furthermore, current limitations and challenges of functional separator design in LMBs, as well as future research directions, are considered.

## 2. Mechanically Strengthened Separator Fabrication

Strengthening separators has been deemed an effective strategy to block the growth of Li dendrites and prevent the short-circuit of LMBs. However, the Li dendrite is sharp and has a high Young’s modulus to pierce commercial separators such as polypropylene (PP) or polyethylene (PE) films [38]. Naturally, designing separators with a Young’s modulus greater than 6 GPa is a straightforward and convenient strategy to protect the separator from punctures [39,40].

**Figure 1:**
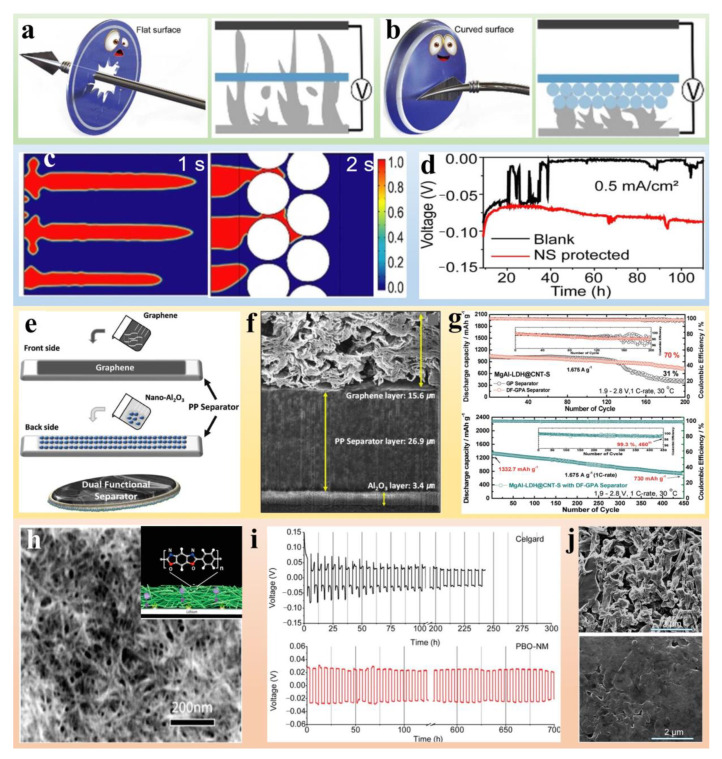
(**a**,**b**) Schematic diagram of Li dendrite blocking behavior with flat/curved shield. (**c**) Finite element simulations: Li dendrite growth against blank separators (**left**); Li dendrite growth against SiO_2_ modified PP separators (**right**). (**d**) Voltage profiles of cells with NS protected and blank separators at 0.5 mA cm^−2^ [41]. Reprinted with permission from Ref. [41]. 2020 John Wiley and Sons. (**e**) Schematic diagram of the synthesis process of the dual-functional graphene/PP/Al_2_O_3_ (DF-GPA) separator. (**f**) Cross-sectional SEM images of graphene/PP/Al_2_O_3_ (DF-GPA) separator. (**g**) Corresponding discharge capacity retentions and cell efficiencies during 200 cycles (**top**) and long-term cycling stability results of a cell based on a MgAl-LDH@CNT-S cathode and a DF-GPA separator (**bottom**) [42]. Reprinted with permission from Ref. [42]. 2017 John Wiley and Sons. (**h**) SEM images of a PBO-NF network, the insert is schematic diagram of Li anode. (**i**) Voltage-time charts at a fixed current density of 0.38 mA cm^−2^ using Celgard 2400 (**top**) and PBO-NM (**bottom**). (**j**) SEM images of Li electrode in contact with Celgard 2400 after 240 h cycling (**top**) and PBO-NM after 700 h cycling (**bottom**) [43]. Reprinted with permission from Ref. [43]. 2016 American Chemical Society.

Coating commercial separators with rigid layers is recognized as a promising way to elevate the puncture strength of the separator. Inorganic materials possessing strong mechanical properties can be coated on the surface of commercial separators to improve the separator strength. Inspired by the shield design, Zhu et al. [41] proposed inhibiting Li dendrite growth by designing a nano-shield separator consisting of SiO_2_ nanoparticles with 500 nm diameter and commercial PP film (Figure 1a,b). By combining theoretical calculations and experiments, they found that a carved shield with a radius comparable to that of the puncturing tip can efficiently distribute interfacial stresses and mitigate short-circuits in LMBs (Figure 1c). Further, the effect of the nano-shield was attributed to a shift in the growth orientation of the Li dendrites and the tortuous growth pathway induced by the SiO_2_ nanoparticles coating. At a current density of 0.5 mA cm^−2^, the voltage plateau of the blank separator abruptly dropped from around 60 mV to roughly 10 mV after about 23 h, indicating that the cell was internally shortened by Li dendrites (Figure 1d). The cell with the nano-shield protected separator achieved a battery life of more than 110 h without short-circuiting, which is approximately five times longer than that of the cell with the conventional blank separator (Figure 1d).

However, due to the ionically insulating properties of some inorganic materials, the transport of Li ions maybe slowed down by the dense and stiff layers on the separator. In order to maintain both the mechanical barrier effect and transport rate of Li ion, Sun et al. [42] used a dual-functional graphene/PP/Al_2_O_3_ (DF-GPA) separator, in which compact graphene and Al_2_O_3_ powder were coated on each side of the separator (Figure 1e,f). The developed DF-GPA plays dual roles in LMBs. First, graphene can serve as strongly conducting layer for smooth electrons and Li ion transportation. Secondly, the Al_2_O_3_ coating layer on the other side provides a superior mechanical property to minimize the risk of short-circuit caused by the penetration of Li dendrites. Consequently, the Li–S cell with a DF-GPA separator harvested a discharge capacity of 730 mA h g^−1^ after 450 cycles at a current rate of 1 C (Figure 1g), exhibiting an improved cycling stability compared with the normal graphene/PP composite separator.

Modification of rigid layers on separator surfaces is an effective approach to suppress Li dendrite growth. However, if an extreme volume change occurs in the electrode, the rigid layer may be peeled off. Moreover, the coating layer would increase the weight of the separator and thus decrease the energy density of LMBs. Therefore, constructing integrated separators is considered a more promising strategy in terms of reducing energy density loss. In this regard, high-modulus polymers are suitable for the fabrication of flexible and high-strength separators that can prevent the growth and penetration of Li dendrites. Sun et al. [43] fabricated a thin nanoporous network from exfoliated poly(p-phenylene benzobisoxazole) (PBO) nanofibers through a simple blade casting process (Figure 1h). The nanoporous membranes (NMs) displayed superior mechanical properties with an ultimate strength of 525 MPa and a high Young’s module of 20 GPa. Symmetric Li|Li cells with Celgard 2400 and NMs were tested to compare the battery life with two distinct separators. The voltage plateau in Celgard 2400 cells decreased from 0.031 V to 0.024 V after 200 h, indicating that the separator was slowly penetrated by Li dendrites (top of Figure 1i). As for the PBO-NM cell, the steady voltage profile was maintained at 0.025 V from the beginning to 700 h, which suggests an effective suppression of Li dendrite growth (Figure 1i bottom). Moreover, dendritic Li appeared on the mossy electrode surface after 230 h of cycling with Celgard 2400, while a smooth and flat Li surface was observed after 700 h of cycling with PBO-NMs (Figure 1j), which again substantiates the ability of PBO-NMs to depress Li dendrite growth.

Stiff and tough polymers are attractive separator materials for the construction of safe and long-life LMBs. However, the electrochemical stability and resistance to solvent dissolution pose a great challenge to the practical application of such separators. More research efforts should be devoted to improving the durability of functional separators.

## 3. Functional Separator Construction towards Regulated Li Ion Deposition

The inhomogeneous deposition of Li ions is prone to result in nucleation and growth of Li metal at high Li-ion concentration spots, leading to Li dendrite formation and subsequent penetration of separators. Regulating Li ion deposition for homogeneous nucleation can effectively address this challenge. Therefore, the construction of uniform nucleation sites on separator surfaces is another tractable and effective method to suppress the growth of Li dendrites [44]. Modified separators with lithiophilic metals [45,46,47], inorganic/heteroatom-doped carbon composite materials [48,49], and 3D architectures with confined spaces [50,51] may provide rapid and uniform diffusion pathways for Li ion transportation and achieve homogeneous Li ion flux on the surface of electrodes, leading to a dendrite-free uniform lithium deposition.

**Figure 2:**
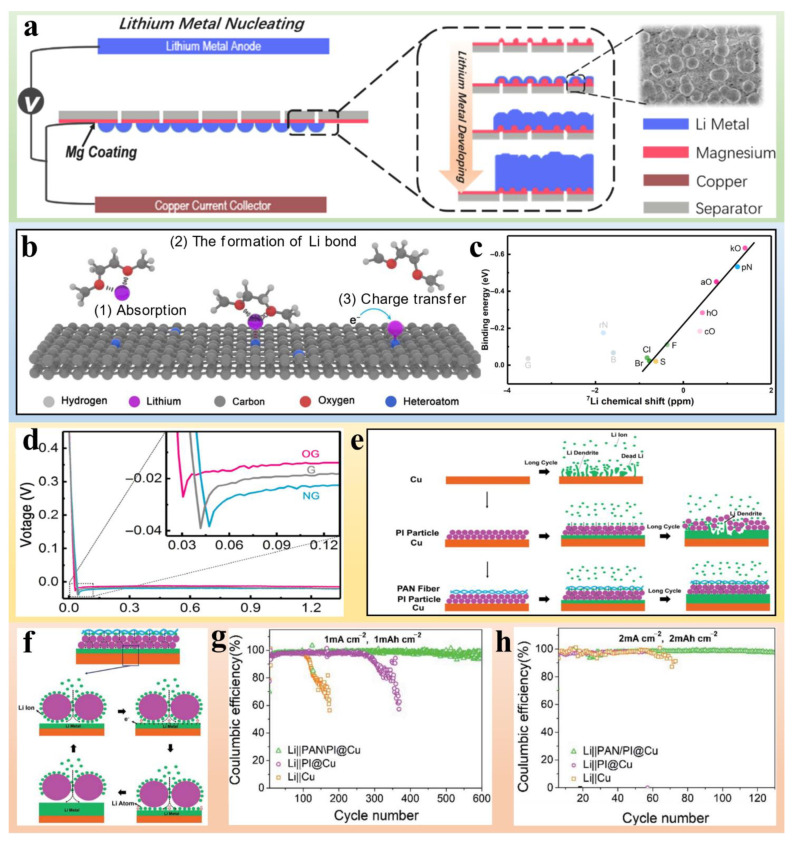
(**a**) Schematic diagram of electrodeposition with a Mg-coated separator [52]. Reprinted with permission from Ref. [52]. 2018 Elsevier. (**b**) Schematic representation of the Li nucleation on carbon materials. (**c**) The correlation between Li atom binding energy theoretical Li chemical shift. (**d**) Voltage-time curves during Li nucleation at 0.50 mA cm^−2^ on pristine graphene (G), nitrogen-doped graphene (NG), and oxygen-containing graphene (OG) electrode [53]. Reprinted with permission from Ref. [53]. 2019 American Association for the Advancement of Science. (**e**) Schematic diagrams of Li deposition behaviors on different substrates. (**f**) Schematic diagram of the dendrite-free mechanism. Comparison of Coulumbic efficiencies of Li|Cu, Li|PI@Cu and Li|PAN/PI@Cu cells (**g**) at 1 mA cm^−2^, and 1 mA h cm^−2^ and (**h**) at 2 mA cm^−2^, and 2 mA h cm^−2^ [56]. Reprinted with permission from Ref. [56]. 2020 The Royal Society of Chemistry.

As for functional separators, lithiophilic metal particles decorated on separators can introduce nucleation sites for Li ions. Song et al. [52] to proposed the deposition of magnesium (Mg) nanoparticles on a single-side of separators by magnetron sputtering to suppress the Li dendrite growth (Figure 2a). The metal Mg particles were chosen for the construction of the lithiophilic separator due to the large solid solubility between the metals Li and Mg. The lithiophilic Mg can considerably reduce the Gibbs free energy for Li electrodeposition, which means that the uniform Mg layer can serve as the heterogeneous nucleation site and guide the even formation of Li metal. The Li|LiCoO_2_ full cell with the Mg-coated separator showed elevated capacity retention, maintaining 80% capacity at 104 mA h g^−1^ after 400 cycles. On the contrary, the cell with the blank separator exhibited a rapidly fading capacity of 75.9 mA h g^−1^ after 330 cycles, which is only 60.2% of the initial capacity. Lithiophilic metal modified separators also face a huge challenge in that the Li metal may deposit at the interface between separators and the modified layer. This causes cracks in the coating layer and fails to suppress the growth of Li dendrites in the long term. Introducing a concentration gradient of nucleation sites in the lithiophilic metal coating layer is expected to solve this problem.

Since Li ions prefer to be absorbed by lithiophilic sites via electrostatic interactions, inorganic materials or heteroatom-doped carbon materials are also efficient in increasing the rate and homogeneity of Li ion flux. Zhang et al. [53] utilized first principles calculations and experimental verification to investigate the lithiophilicity chemistry of heteroatom-doped carbon materials. Twenty species with various dopant forms were modeled to construct heteroatom-doped graphene nanoribbons. It was concluded that the carboxylic group, pyridinic nitrogen, and ketone group exhibited the largest binding energy (Figure 2b,c). To validate the calculated result, Li nucleation overpotential was tested to evaluate the lithiophilicity of three kinds of graphene materials: pristine graphene (G), nitrogen-doped graphene (NG), and oxygen-containing graphene (OG), which had the nucleation overpotential of 22.9, 19.4, 15.0 mV, respectively, indicating that OG processed the lowest nucleation energy barrier and the best lithiophilicity (Figure 2d). More significantly, separators modified with carbon materials can be applied to reduce the polysulfide shuttle in Li-S batteries. Porous carbon materials can not only can strengthen the physical absorption of long-chain polysulfides, but also can effectively enhance the chemical affinity of polysulfides due to their polar functional groups. Nitrogen-doped carbon was developed to modify PP separators by Balach et al., and promoted the absorption of long-chain polysulfides because of the unique physical and interfacial chemical properties of doped-carbon layer. The deterioration rate at 0.5 C for a cycle stability of more than 1200 cycles was only 0.037%, exhibiting an enhanced cycle stability and capacity retention [54]. Some inorganic materials with rich inherent defects, such as TiO_2_, can accelerate the redox kinetic of polysulfides. Xiao et al., designed separators modified with a nonpolar conductive physical layer (graphene) and polar chemical absorption layer (TiO_2_), which demonstrate a very high reversible specific capacity beyond 1000 mA h g^−1^ over 300 cycles at 0.5 C [55].

Additional materials with polar groups can also regulate Li ion deposition through electrostatic interactions. Han et al. [56] developed polyacrylonitrile fiber/polyimide sphere (PAN fiber/PI sphere) double-layer coating to guide uniform deposition of Li ions and suppress Li dendrites growth (Figure 2e). The mechanism of dendrite-free deposition on a separator with a PAN fiber/PI sphere is shown in Figure 2f. The lone pair of electrons of oxygen in the oxygen-containing functional groups (C=O) of PI exhibit considerable affinity for Li ions and may evenly adsorb Li ions on their surfaces. When Li ions obtain an electron and are reduced into the Li atom, the interaction between Li atoms and PI spheres become weaker, causing the uniform deposition of Li metal on the surface of electrode. Li|PAN/PI@Cu (where “|” and “@” mean “countering” and “coating”, respectively) has a steady CE and shows better cycling stability than Li|Cu and Li|PI@Cu, exhibiting a high CE of 98% after 500 cycles (Figure 2g). PAN/PI@Cu still delivers a high CE of 97.3% for 130 cycles even at a raised current density of 2 mA cm^−2^ with a deposition capacity of 2 mA h cm^−2^ (Figure 2h).

Designing the coating layer of separators with a polar group is a useful strategy to regulate the deposition of Li ions and suppress Li dendrite growth. However, if the electron conductivity of the anode is lower than that of the coating layer in functional separators, the Li ion might be deposited in the coating layer instead of the anode matrix. Therefore, it is advisable to pay attention to problems concerning matching between the anode matrix and coating materials while constructing functional separators modified with polar groups.

## 4. Flame-Retardant Separator Design

**Figure 3:**
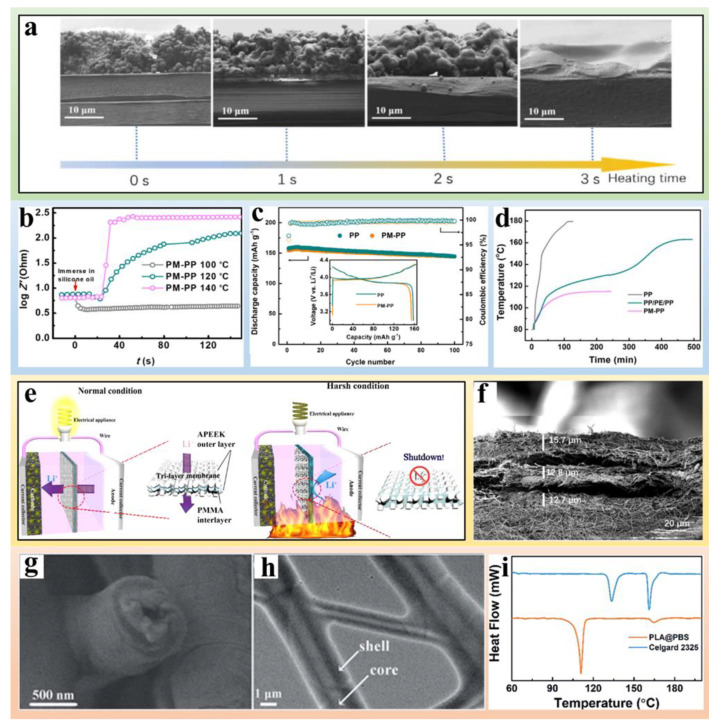
(**a**) Morphological and structural changes of the PM coating layer with heating time in a 110 °C PC bath. (**b**) Log Z-t plots of the SS/separator/SS coin cells using commercial PP/PE/PP separator. (**c**) Current density for the charge-discharge process at 0.5 C. (**d**) Adiabatic T-t curves of the LiCoO_2_|Li coin cells using different separators in short circuit tests [59]. Reprinted with permission from Ref. [59]. 2019 Elsevier. (**e**) Conceptual plan of the trilayer nonwoven membrane. (**f**) Cross-section of the NW-CA/P/CA membrane avulsed using adhesive tape [61]. Reprinted with permission from Ref. [61]. 2018 Elsevier. (**g**) SEM and (**h**) TEM images of the PLA@PBS separator. (**i**) DSC curves of the PLA@PBS and Celgard 2325 separators [63]. Reprinted with permission from Ref. [63]. 2017 The Royal Society of Chemistry.

To guarantee battery safety, thermally responsive separators need to be constructed to cope with elevated temperatures and thermal runaway. Separators with thermal response are developed with a view to thermal shutdown and flame-retardation. Thermal shutdown separators constantly incorporate thermoplastic polymer particles, which would melt and form an insulating barrier to inhibit Li^+^ transport, interrupting the battery reactions as the temperature reaches a critical value [57,58]. PE microspheres with low-density are commonly employed as a shutdown layer benefiting from their enormous surface areas, which allow them to respond quickly to temperature changes in batteries, and their abundance of microporous channels for the movement of electrolyte. Zhong et al. [59] coated a thin layer of low-density polyethylene microspheres (PM) onto commercial PP separators and investigated their thermal response behaviors. A PM layer with a porous structure profited from normal charge-discharge reactions at ambient temperature, but began to melt at 110 °C and collapsed to form dense protected layers for interrupting the battery reaction after 3 s (Figure 3a). PM/PP separators have rapid thermal shutdown behaviors with impedance starting to increase at 120 °C in 20 s and then rapidly rising within 5 s at 140 °C (Figure 3b). What is more, the electrochemical performance of the cell with PM/PP separator is almost same as that of the cells with the PP separator (Figure 3c). Compared to the blank PP and PP/PE/PP separators, the PM/PP separator is effective in controlling the temperature rise of the Li battery by timely melting of the PM coating layer (Figure 3d).

A multilayer structure is also employed to construct separators with shutdown properties. Multilayer separators are always composed of an interlayer with a lower melting temperature and outer layers with higher thermal stability. When the temperature rises, the interlayer fuses and merges with outer layers to form a pore-free layer, cutting off the channel of Li^+^ transport [60]. Xu et al. [61] developed a tri-layer membrane by sandwiching a poly(methyl methacrylate) interlayer between amido functionalized poly(ether ketone) outer layers (APEEK) to protect the Li battery from thermal runaway (Figure 3e). The fusible interlayer melts and blocks the pore of non-woven crosslinking APEEK/PMMA/crosslinking APEEK (NW-CA/P/CA) tri-layer membrane above 110 °C (Figure 3f), which prevents the transfer of Li ions between the electrodes and suppresses the electrode reactions.

For multilayer separators, compatibility issues and weak adhesion pose great challenges for different layers. In addition, inhomogeneous coatings can affect Li^+^ migration rate and thermal shutdown performance. To address the above issues, coaxial electrospinning is a tractable and well-established technique for fabricating core-shell-structured membranes with shutdown properties [62]. Taking advantage of this method, Cao et al. [63] successfully fabricated poly(lactic acid)@poly(butylene succinate) (PLA@PBS) as a shutdown separator (Figure 3g,h). PLA is used as the core material due to its excellent thermal stability and mechanical strength, and PBS is used as a shell material due to its strong affinity for liquid electrolytes and appropriate melting temperature. Compared to differential scanning calorimetry (DSC) results between PLA@PBS separators and the Celgard 2325 separator, PLA@PBS separators exhibited lower shutdown temperatures at 110.5 and 165.0 °C, reducing the probability of thermal runaway (Figure 3i).

**Figure 4:**
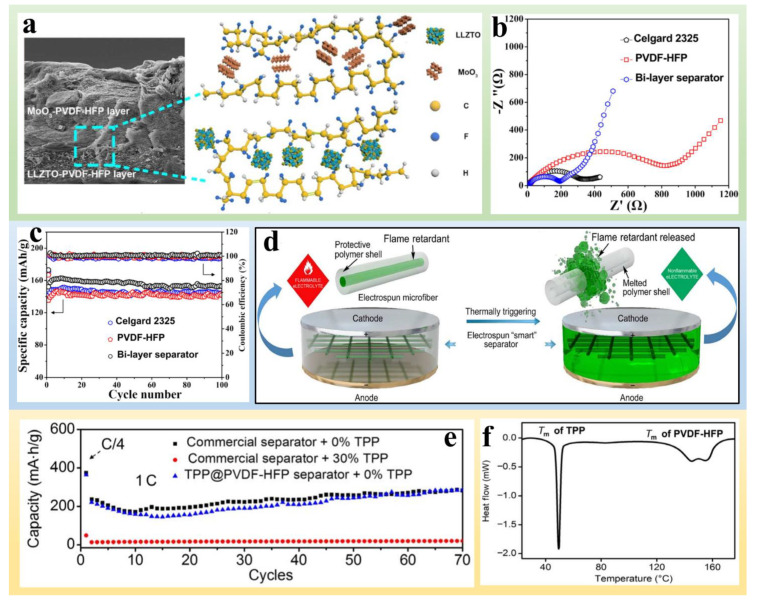
(**a**) Cross-sectional SEM images of the bilayer separator and a schematic illustration of the bilayer structure. (**b**) EIS of Li/LFP cells with Celgard 2325, PVDF-HFP, and bilayer separators. (**c**) Cyclic performances of Li/LFP cells assembled with Celgard 2325, PVDF-HFP, and a bilayer separator [71]. Reprinted with permission from Ref. [71]. 2019 American Chemical Society. (**d**) Schematic of the “smart” electrospun separator with thermal-triggered flame-retardant properties for Li-ion batteries. (**e**) Electrochemical performances of a graphite anode using different combinations of separators and electrolytes. (**f**) DSC of the TPP@PVDF-HFP separator [69]. Reprinted with permission from Ref. [69]. 2017 American Association for the Advancement of Science.

Flame-retardant separators incorporated with extinguishing agents can effectively prevent the combustion caused by thermal runaway and improve the safety of LMBs [64,65,66]. Organic and inorganic materials, such as triphenyl phosphate (TPP), metal hydroxides, Sb_2_O_3_, MoO_3_, are commonly used as extinction agents [67,68,69]. Among them, the metal hydroxides Mg(OH)_2_ and Al(OH)_3_ are the most common inorganic flame retardants, and efficiently reduce the surface temperature of the combustible materials and inhibit the combustible gas by releasing lots of water at elevated temperature and absorbing plenty of heat when combined with water vapor. Organic materials are mostly halogen- or phosphorus-based molecules that exhibit flame retardation by suppressing chain reactions and flame propagation through radical scavenging processes or physical isolation mechanisms [69,70]. He et al. [71] constructed a bilayer separator by incorporating MoO_3_ and Al-doped Li_6.75_La_3_Zr_1.75_Ta_0.25_O_12_(LLZTO) (Figure 4a). Combustion was considerably suppressed with interfacial adhesion of MoO_3_ and LLZTO to poly(vinylidenefluoride-hexafluoropropylene) (PVDF-HFP) by abundant hydrogen bonds and van der Waals forces of the bilayer separators. LLZTO shows excellent Li-ion conductivity, and MoO_3_ exhibits remarkable flame-retardant capabilities by lowering the generation of volatile hydrocarbon species and flammable benzene species. A Li|LiFePO_4_ cell with such bilayer separator also exhibited lower charge-transfer resistance and faster reaction kinetics compared to those with Celgard 2325 and PVDF-HFP (Figure 4b). The discharge capacity could reach 162 mA h g^−1^ at 0.5 C after 100 cycles with a capacity retention of 95% and high average CE of near 100%, further demonstrating the superiority of the bilayer separator to the blank separator in LMBs (Figure 4c).

Because some flame retardants can increase electrolyte viscosity and thus reduce the ionic conductivity of electrolytes, thermal-sensitive polymers have been adopted as a protective layer to prevent the exposure of flame retardants to the electrolyte and sustain the ionic conductivity [72]. Cui et al. [69] developed a microfiber with a core-shell structure by electrospinning, where the triphenyl phosphate (TPP), a common organophosphorus-based flame retardant, was the core and PVDF-HFP was the shell (Figure 4d). When an Li battery suffers from thermal runaway, the shell melts and releases the flame retardant (TPP) due to the increasing temperature, suppressing combustion of the highly flammable electrolytes. Compared to commercial separators with compositing 30% TPP, the TPP@PVDF-HFP core-shell structure avoids the negative effects of TPP on the electrochemical performance of the anode and shows excellent capacity when equipped with commercial separator (Figure 4e). More importantly, the PVDF-HFP melts and releases TPP when the temperature rises to 150 °C (Figure 4f), and the encapsulated TPP (~100%) is abruptly released into the electrolyte upon heating up to 160 °C.

## 5. Summary and Outlook

The latest developments on functional separators for long-life and safe Li metal batteries have been summarized and discussed in this minireview, including mechanically strengthened separator fabrication, functional separator construction towards regulated Li ion deposition, and flame-retardant separator design. The basic mechanism for mechanically strengthened separator fabrication is attributable to the mechanical barrier effect with higher puncture strength, strong enough to bend the tip of the Li dendrite. Functional separator construction towards regulated Li ion deposition can construct even lithiophilic sites for uniform nucleating and growth. Flame-retardant separator design aims at controlling thermal runaway by forming dense separators to interrupt battery reactions or releasing flame retardants to prevent burning. Although various strategies have been applied to improve the functional separators for practical applications of LIBs, there are still some challenges that need to be addressed.

(1) Most of the reported functional separators have been developed with top-down strategies such as the tap-casting method, which leads to a lack of control over the thickness and the homogeneous distribution of different components. More elaborate bottom-up strategies should be designed to develop uniform and homogenous separators.
(2) To minimize sacrifice in both volumetric and gravitation energy density, the thickness of the modification layer should be controlled at <1 µm (<5% in thickness compared to commercially separator such as Celgard 2325) and its density should be reduced as low as possible. In this regard, novel nanostructured nanomaterials, especially 2D graphene and boron nitride, are good options.
(3) To avoid peeling of the modification layer from the separator during Li plating/stripping cycles, molecular and structural designs to improve the flexibility and elasticity of the modification layer, as well as its adhesion on the separator, are highly desirable.
(4) To help eliminate battery thermal runaway caused by Li dendrites and improve battery safety, the thermal conductivity of the separator should be further improved for better battery heat dissipation; few studies have focused on this.
(5) The “activation” of functional separators should be understood in depth. Some of the designed separators are not electrochemically inert, and they may absorb Li^+^, or even react with electrolyte or Li metal electrodes, especially under complex electrochemical conditions. These “activations” (absorption and reaction) together with the influence on battery performance should be carefully studied.
(6) The performance of the designed separators should be evaluated under practical conditions. The areal current density and capacity for Li|Li symmetric cells and Li metal full cells should be higher than 3 mA cm^−2^ and 3 mAh cm^−2^, respectively, because the area capacity of commercial energy-type LMBs is >3 mAh cm^−2^ and the discharging/charging current is >3 mA cm^−2^ based on 1 C rate. The electrolyte amount should be controlled at <10 µL mAh^−1^ (requirement of lean electrolyte), and the capacity ratio of Li metal anode and cathode should be <5 to maintain high volumetric energy density.

## Data Availability

Not applicable.
